# A systematic review of the measurement properties of self-care scales in nurses

**DOI:** 10.1186/s12912-023-01450-2

**Published:** 2023-08-28

**Authors:** Nahid Rajai, Abbas Ebadi, Leila Karimi, Seyedeh Azam Sajadi, Akram Parandeh

**Affiliations:** 1 Student Research Committee, Nursing Faculty, Baqiyatallah University of Medical Science, Tehran, Iran; 2 Behavioral Sciences Research Center, Life style Institute, Nursing Faculty, Baqiyatallah University of Medical Sciences, Tehran, Iran; 3 Nursing Management Department, Nursing Faculty, Aja University of Medical Sciences, Tehran, Iran; 4 Medicine, Quran and Hadith Research Center, Nursing Faculty, Baqiyatallah University of Medical Sciences, South Sheikh Bahai St, Mollasadra St., Vanak Square, Tehran, Iran

**Keywords:** Nurse, Self-care, Scale, Systematic review, COSMIN checklist

## Abstract

**Background:**

Self-care is a necessary measure against occupational injuries of nurses and improves nursing performance at the bedside. Nurses have different scales to measure self-care, and researchers are confused about choosing valid and reliable scales. This systematic review aimed to evaluate the measurement properties of self-care scales in nurses to identify the best available scales.

**Methods:**

Four databases (PubMed, Web of Science, SCOPUS, and ProQuest) were systematically searched, with no date limiters, until 9 Jun 2023. A manual search was performed with Google Scholar and the reference list of articles to complete the search. Studies aiming to develop or determine the measurement properties of self-care in nurses were included. Based on Consensus-Based Standards for the Selection of Health Measurement Instruments, the methodological quality of the studies was determined, and the result of each study on a measurement property was rated (sufficient, insufficient, or indeterminate). The quality of the evidence was graded using a modified Grading of Recommendations Assessment, Development, and Evaluation approach (high, moderate, low, or very low). These processes were used to make recommendations and identify the best scale to assess self-care in nurses.

**Results:**

Out of 8601 articles, six articles with five different scales were included. Only internal consistency was reported across all scales. Criterion validity, measurement error, responsiveness, feasibility, and interpretability, were not reported in any of them. Content validity was reported only in two studies with inconsistent results and low-quality evidence. None of the scales had methodological quality with a rating of very good and sufficient high-quality evidence for all measurement properties.

**Conclusions:**

None of the scales is strongly recommended to measure self-care in nurses. Only the Professional self-care scale is temporarily recommended until their quality is assessed in future studies. Considering that the content of the examined scales does not meet all the professional self-care needs of nurses, designing a valid, reliable, and specialized scale for nurses is needed.

**Supplementary Information:**

The online version contains supplementary material available at 10.1186/s12912-023-01450-2.

## Background

Nurses face occupational hazards in their work environment every day, which exposes them to health risks. For example, night shifts, sleep deprivation, exposure to violence, contagious diseases, hazardous chemical materials or radioactive rays, fatigue, stress, vigorous activity, prolonged standing, etc. [1]. that lead to the occurrence of diseases including sleep disorders [2], cancer [3], cardiovascular diseases [4] and musculoskeletal injuries [5].

In this regard, self-care is a key solution to preventing occupational diseases and injuries. Self-care was first proposed by Dorota Orem, a nursing theorist, between 1959 and 2001. Self-care is a purposeful and conscious act that people do to maintain their life, health, and well-being [6]. Self-care enables nurses to maintain their health and progress in their work despite job stress [7], reduces job burnout [8], increases the quality of work life, improves the quality of the care they provide, and finally maintains patient safety [9]. The importance of self-care in nurses is so much that it has been introduced as one of the ethical codes of nursing by the American Nursing Association (ANA) [10].

Despite the importance of self-care, nurses often neglect it [11]. Also, this issue has not been considered in the nursing curriculum [12]. But after the covid-19 pandemic, which was associated with the death and disability of many healthcare workers, especially nurses with the most contact with patients [13], the concept of nurses’ self-care became more important. Also, the attention of researchers increased to measure the levels of self-care in nurses and the related factors [14, 15] or to investigate the effect of various interventions in promoting it [16]. However, researchers are faced with a wide range of self-care scales. About 42 self-care scales have been designed for various populations and health conditions [17]. For this reason, there is often confusion in choosing the suitable scale. Because in addition to the multitude of scales, the evidence shows that some are not valid and reliable [17] and do not cover all the self-care needs of nurses according to the nature of their work. However, regardless of these challenges, researchers have repeatedly used these scales. While not using the appropriate measurement scale leads to wasted resources and unreliable results and has ethical issues [18].

To evaluate the validity and reliability of a scale, one must assess the measurement properties (e.g., structural validity, responsiveness, etc.). A systematic review of measurement properties of scales is a useful way to select the best scale to measure a particular phenomenon [19]. These studies can also identify gaps in knowledge regarding the measurement properties of existing scales, which can be used to design new measurement properties [20]. Also, the Consensus-Based Standards for the Selection of Health Measurement Instrument (COSMIN) is a standardized guideline for designing scales and assessing the methodological quality of studies on measurement properties [21]. Using this guideline, the examined scales can be placed in three categories: A- scales that are the most suitable for use, B- scales that can be used temporarily but need more studies and C- scales that should not use [22].

Evaluation of scales through these studies is increasing. In concern to self-care scales, studies including evaluation of self-care scales in hypertensive patients [23], diabetic patients [24], or healthy people [17] have been investigated. In all of these studies, the investigated scales had serious problems in measurement properties. To our knowledge, no systematic review has been conducted on nurses’ self-care scales. This study is thought to help researchers select the most suitable scales.

## Method

### Aim

This systematic review aimed to evaluate the measurement properties of self-care scales in nurses using the COSMIN methodology to identify the most suitable available scales.

### Study design

This study follows the COSMIN Methodology for Systematic Reviews of Measurement properties of Patient Reported Outcome Measures (PROMs) [22, 25], the COSMIN methodology for evaluating the content validity of PROMs [26], COSMIN Risk of Bias checklist for systematic reviews of PROMs [27] and Preferred Reporting Items for Systematic Reviews and Meta-Analyses (PRISMA) guideline [28].

### Search methods

#### Information sources

One author (NA), with the assistance of an expert in medical library and information science, developed a search strategy and conducted a literature search.The SCOPUS, ProQuest, ISI Web of Science, and PubMed databases were used to conduct time-free searches for publications through 9 Jun 2023. Additionally, Google Scholar was used as a comprehensive and scientific search engine for manual searching and accessing similar papers that weren’t available in other databases to enhance the electronic search. Also, we looked through the reference lists of all featured papers to see if any additional ones would qualify.

#### Eligibility criteria

Peer-reviewed, full-text, accessible English and Persian articles with the subjects “develop” or “determine the measurement properties of nurses’ self-care scales” were considered eligible. This study excluded conference papers, unpublished manuscripts, dissertations, and thesis, letters to the editor, book chapters, reviews papers, qualitative papers, and articles that did not evaluate the measurement properties.

#### Strategy of search

According to COSMIN recommendations about the keywords, four main factors: (1) the construct (self-care); (2) the population (nurses); (3) the type of scale (patient-reported outcome measures (PROM) or self-report); and (4) the measurement properties of interest (i.e., reliability, validity, and responsiveness) were used [22]. Also, we used the entry terms below of each concept in the MeSH and combined them with Boolean operators AND and OR. We also find free keywords by asking the specialists. Then, the applicable search strategy in PubMed was defined for other databases. Finally, Keywords of “self-care; self-care deficit; self-care agency; self-care requisites; personal self-care; organizational self-care; care of self; care of the self; care for themselves; mindful self-care; themselves-care; professional self-care; nurse; nursing staff; nursing personnel; nursing assistants; scale; instrument; tool; validation; psychometric; inventory; checklist; questionnaire; assessment; measurement; evaluation” were selected. In Additional file 1, each database’s search syntax is described.

#### Selection process of studies

Endnote Software, version X9.1.19.0.0.12062, was used to manage the searched articles. After eliminating duplicate entries, one of the authors (NR) reviewed the titles and abstracts of the articles to see whether they met the inclusion criteria. The entire texts of the articles chosen in the previous phase were then retrieved, and their suitability was independently assessed by two authors (NR and AP). When there was a dispute over an article’s selection, the fourth author (SAS) was consulted.

### Appraising quality

In this study, with the use of the COSMIN guideline user manual for systematic scales reviews [22], the methodological quality of single studies on measurement properties and the quality of the scales themselves (i.e.,their measurement properties) were assessed separately. Then the results of these two types of quality assessment were combined. Two authors (NR and SAS) did data extraction from studies and assessments independently, and in case of doubt, the third author was consulted (AE).

#### Assessing the methodological quality of studies

At first, the quality of the studies on scale development (Item generation) and the content validity of each scale (In terms of three features of relevance, comprehensiveness, and comprehensibility) were checked, using manual user of COSMIN methodology for assessing the content validity of PROMs [29]. Then, the methodological quality of studies on other measurement properties (structural validity, internal consistency, cross-cultural validity, reliability, Measurement error, criteria validity, hypotheses testing for construct validity, and responsiveness) was assessed based on the COSMIN Risk of Bias Checklist. The four-point rating system ) very good, adequate, doubtful, or inadequate quality( and the ‘worst score counts’ principle were used to score the articles [25, 27].

#### Assessing the quality of measurement properties of the included scales

In this section, data on attributes of scales, such as characteristics of included populations and results related to measurement properties, was extracted from the studies by two authors (NR, SAS). Then, based on the user manual of COSMIN guideline, the results of measurement properties were scored against the updated criteria for good measurement properties as either sufficient (+), insufficient (–), or indeterminate (?). When the measurement properties of a scale have been examined in multiple studies, the results were qualitatively summed up and rated again (Overall rating**)** according to the 75% agreement rule as sufficient (+), insufficient (-), inconsistent (±), or indeterminate (?) [25].

The content validity property was scored in terms of three criteria of relevance, comprehensibility, and comprehensiveness, using criteria for good content validity. According to the guidelines, scoring was done both for the studies on scale development and the studies that separately examined the content validity of these scales (content validity studies). Also, the reviewers (research team) also reviewed and scored the scales items separately [29].

#### Grading the quality of evidence of measurement properties

Finally, using a modified Grading of Recommendations Assessment, Development, and Evaluation (GRADE) technique for systematic reviews, the quality of the summarized evidence was rated (high, moderate, low, or very low). The criteria used in this approach to determine the quality of evidence were the risk of bias in the methodological quality of the studies, unexplained inconsistency of results across studies, the total sample size of the studies (imprecision), and the evidence from different samples than the samples of interest in the review (indirectness) [25].

It should be noted that in grading the quality of evidence of content validity, the risk of bias is related to the quality of the PROM development study or the quality of additional content validity studies. Also, in this property, the criterion of imprecision wasn’t considered because PROM development and content validity studies were assessed in qualitative research.

### Categorizing the investigated scales

**Fig. 1:**
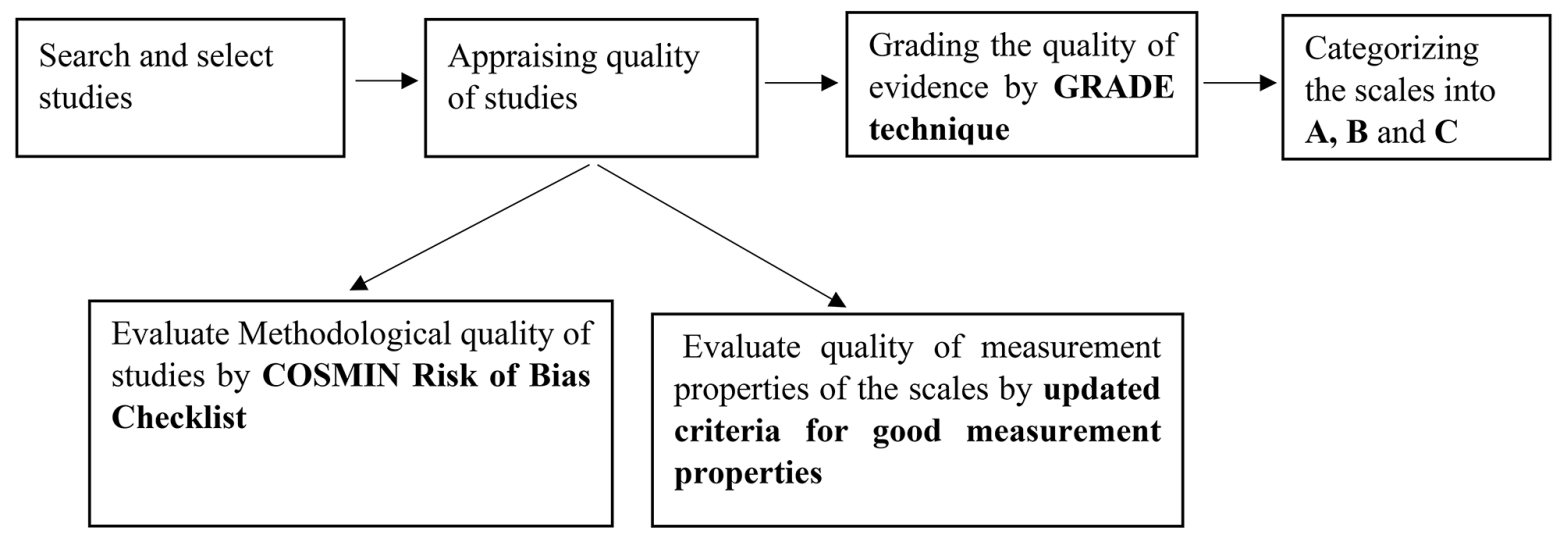
Study design according to COSMIN guidline [25]

According to the results of the previous stages, the scales were categorized as A, B, and C. Group A scales have trusted results and are recommended for use. In B group, scales have the potential to be approved for usage, but more study is needed to determine their quality. It is not recommended to employ a scale with the classification “C”. Also, the COSMIN guideline suggests that the scales can be better selected by collecting information on the interpretability (e.g., floor and ceiling effects, minimal important change (MIC), percentage of missing items, etc.) and feasibility (e.g., completion time, cost, copyright, etc.) of the scales. Especially when the scales are in the same categories regarding the quality of evidence [25]. The study design is shown in Fig. 1.

## Results

### The systematic literature search result

**Fig. 2:**
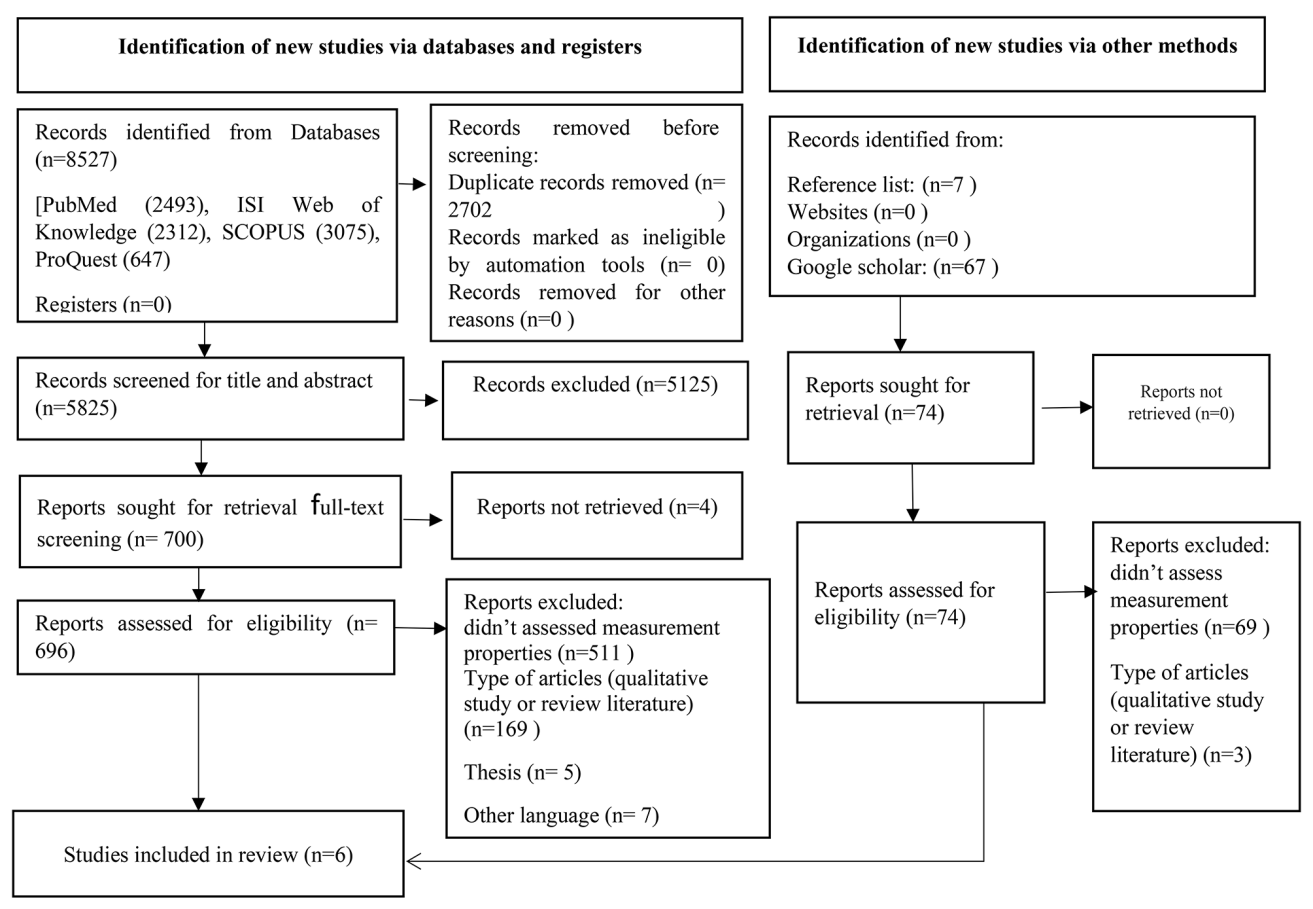
PRISMA 2020 flow diagram [28]

In general, 8601 records were found in the search via databases and other methods. After removing duplicates and screening in terms of title, abstract, and then full text, six eligible articles and five scales were included. The details of the selection process of the articles are given in Fig. 2.

### Characteristics of included studies and scales

The characteristics of the studies and scales are reported in Table 1, and characteristics of study populations involved in developing and validating eligible scales are reported in Table 2. All of the scales included are paper-based questionnaires and self-reported. In the examined scales, the number of items was between (9–75). Among scales, only the Professional Self-Care scale (PSC) was specifically designed to examine self-care in nurses, and the rest were general self-care scales, some of which have been assessed in the population of nurses, in secondary studies. The extracted data related to the measurement properties of the scales, in each study are also reported in Table 1.

**Table 1:** Characteristics of the scales used to measure self-care of nurses and their studies

Scales	Authors (year)	Country	Response options / (no. items)	Dimensions	Measurement properties
1. Self-Care Behaviors of Nurses Scale (SCBN)	Sabourian Jouybari et al. 2016 [14]	Iran	Likert (34 items)	Nutritional/Physical/Individual Health/Psycho-Social/Belief	- *Qualitative content validity/Internal consistency*: Cronbach’s alpha: 0.88
2. Professional Self-Care Scale (PSC)	Galiana et al. 2015 [29]	Spain	Likert (9 items)	Physical/Internal/Social	- *Construct validity*: CFA: Adequate fit: (χ2 (24, N = 385) = 140.66, p < 0.01; CFI = 0.91; GFI = 0.93; SRMR = 0.09; and RMSEA = 0.10). All factor loadings and correlations were statistically significant (p < 0.01) /*Internal consistency*: Reliability indices for the physical, internal and social dimensions (Rho and GLB of 0.64, 0.90, and 0.57, respectively); (Cronbach’s alpha of 0.62, 0.84, and 0.57)/ *Hypothesis test*: Positive correlations among self-care dimensions and awareness, coping with death, compassion satisfaction (p < 0.01) and negative correlation with secondary trauma (p < 0.05) and burnout (p < 0.01))/*Pillai’s criterion*: Combined dependent variables were significantly affected by discipline (F (9, 1074) = 2.00, p = 0.04) and gender (F (3, 371) = 3.19, p = 0.02) of professionals
3. Self-Care Assessment Worksheet (SCA)	Kohli et al. 2020 [30]	India	Likert (70 items)	Physical/Spiritual-Psychological/Mental/Professional/Balance	- *Internal consistency*: Cronbach’s alpha: 0.86
4. Mindful Self-Care Scale (MSC)	Zeb et al. 2022 [31]	Pakistan	Likert (36 items)	Mindful relaxation, physical care/Self-compassion and purpose/Supportive relationships/Supportive structure/Mindful awareness/General	- *Internal consistency*: Cronbach’s alpha of 0.72 and the alpha for the subscales ranged from 0.65 to 0.72.
5. Mindful Self-Care Scale (MSC)	Yang et al. 2021 [32]	China	Likert (24 items) .	Mindful relaxation/ Physical care/Self-compassion and purpose/Supportive relationships/Supportive structure/ Mindful awareness	- *Item Analysis*: CR of 24 items (7.780–19.567); The scores of each item were positively correlated with the total score (r = 0.404–0.740, P < 0.001); Cronbach’s alpha of the translated scale, after deleting each item (0.914– 0.919)/*Reliability*: Split-half reliability (r = 0.770); Test-retest reliability (r = 0.732)/*Internal consistency*: Total Cronbach’s alpha of scale: 0.920; Mindful relaxation: 0.919; Physical care: 0.899; Self-Compassion and Purpose: 0.869; Supportive Relationships: 0.933; Supportive structure: 0.850; Mindful awareness: 0.853/*Content Validity*: I-CVI (0.857–1.000); S-CVI (0.946)/*Construct validity*: EFA: KMO: 0.883, Bartlett test of Sphericity (2 = 9491.909; P < 0.001), the six factors explained 8.685, 9.787,12.185, 13.422, 14.595, and 14.949% of the variance, respectively after the Varimax rotation; CFA: (χ2/df = 2.431, GFI = 0.917, AGFI: 0.905, PGFI:0.703, RMSEA = 0.043, and CFI = 0.96, IFI:0.96, NFI: 0.91, PNFI:0.78, TLI: 0.95)/*Cross-cultural validity*
6. Self-Assessment Questionnaire (SAQ)	Ahmadi et al. 2019 [13]	Iran	Likert (75 items)	Physical health/Mental health/Ability to prioritize/Personal management/Supportive relationships/Being effective in life	- *Quality face and content validity/Internal consistency*: Cronbach’s alpha: 0.94./*Cross-cultural validity*

NR = Not report; CFA: confirmatory factor analysis; EFA: exploratory factor analysis; KMO: Kaiser-Meyer-Oklin; RMSEA: Root Mean Square Error of Approximation; CFI: Confirmatory Fit Index; TLI: Tucker-Lewis Index; NFI: Normed Fit Index; PNFI: Parsimonious normed-of-fit index, CRIs: Composite Reliability Indexes; SOC: Sense of Coherence Questionnaire; CR: Critical ratio; GFI: Goodness-of fit index; AGFI: Adjusted goodness-of-fit index; PGFI: Parsimonious goodness-of-fit index, IFI: incremental fit index; CVR: Content validity ratio; CVI: Content validity index

**Table 2:** Characteristics of study populations involved in the development and validation of eligible scales

Scales	N	Age (year)	Language	Sex (% female)	Professionals’ discipline	Professional experience	Setting
Sabourian Jouybari et al. (SCBN) [15]	100	20–30	Persian	90	Nurse	NR	Cardiac care
Galiana et al. (PSC) [30]	385	NR^*^	Spanish	77.5	Nurse/Nursing assistants/Doctor/Psychologists/Support staff	NR	Palliative care
Kohli et al. (SCA) [31]	134	20–59	Indian	50.7	Nurse/Doctor/Psychologist	1 to 20 Years	Oncology care
Zeb et al. (MSC) [32]	50	> 18	English	65.9	Nurse	> 3 Month	Acute care
Yang et al. (MSC) [33]	510	> 18	China	79.8	Nurse	> 1 Year	Hospice care
Ahmadi et al (SAQ) [14]	310	19–62	Persian	76	Nurse	2 Month to 28 Years	General care

* Not reported

### Content validity of scales

Only two studies reported content validity among the six included studies [30, 33]. The rest of the studies did not report the PROM development or the original scale could not be accessed, despite the researcher’s follow-up. The scoring of the content validity property of these scales is reported in Table 3. In the PSC and Mindful Self-Care Scale (MSC), the target population didn’t involve in the development of the scale. So, the quality of the PROM development of these scales was inadequate. None of the scales had sufficient content validity with high-quality evidence. Overall rating of the result of three aspects of content validity (relevance, comprehensiveness, and comprehensibility) for PSC and MSC showed inconsistent results with low-quality evidence.

**Table 3:** COSMIN methodology for assessing the content validity of self-care scales in nurses

Studies (Scale)	PROM development ^1^	Content validity ^2^	Relevance ^3^				Comprehensiveness ^3^				Comprehensibility ^3^				Content validity rating
			PROM development study	Content validity study	Rating of reviewers	Overall rating ^4^& QoE ^5^	PROM development study	Content validity study	Rating of reviewers	Overall rating ^4^& QoE ^5^	PROM development study	Content validity study	Rating of reviewers	Overall rating ^4^& QoE ^5^	Overall rating ^4^& QoE ^5^
Galiana et al. [30] (PSC)	Inadequate	Doubtful	±	?	+	Low (±)	-	?	+	Low (±)	+	?	+	Moderate (+)	Low (±)
Yang et al. [33] (MSC)	Inadequate	Doubtful	±	±	+	Low (±)	+	-	+	Low (±)	+	+	+	Moderate (+)	Low (±)

^1^Quality of the PROM development (Rating: very good, adequate, doubtful, inadequate); ^2^Quality of content validity studies on the PROM (Rating: very good, adequate, doubtful, inadequate); ^3^Result of the single studies against the criteria for good content validity (Rating: sufficient (+), insufficient (–), or indeterminate (?); ^4^Overall rating of result (Rating: sufficient (+), insufficient (–), inconsistent (±), or indeterminate (?)); ^5^quality of the evidence, using a modified GRADE approach (high, moderate, low, very low)

### The methodological quality of the studies

Six studies with measurement properties were scored for methodological quality using the COSMIN Risk of Bias Checklist (Table 4). Internal consistency was the only property assessed and reported in all studies. Measurement error, responsiveness, and criterion validity were not measured and reported in the studies. All scales in studies were based on a reflective model and were made based on the Classical Test Theory (CTT). The hypothesis test was only assessed in the study of Galiana et al. [30] that used the convergent validity method and had adequate methodological quality.

Structural validity was reported in two studies, which had very good methodological quality due to the report CFA and EFA and sufficient sample size [30, 33]. Methodological quality was insufficient in two studies that examined cross-cultural validity. Because in the study of Yang et al. [33] despite the appropriate approach to analyze the data and adequate sample size, samples were not similar for relevant characteristics except for the group variable (Language). Also, in studies by Ahmadi et al. [14], the inappropriate approach was used to analyze the data with an inadequate sample size (n < 100). Reliability (Test-retest) was reported only in the study of Yang et al. [33] that had inadequate methodological quality. In this study, the time interval between the administrations was long (5 weeks); in the COSMIN guideline, two weeks is suitable.

**Table 4:** Methodological quality of studies using the COSMIN Risk of Bias Checklist

Studies (Scales)	Structural validity	Internal consistency	Cross-cultural validity (Measurement invariant)	Reliability (Test-retest)	Criterion validity	Construct validity (Hypotheses testing)	Measurement error	Responsiveness
Sabourian Jouybari et al. (SCBN) [15]	NR	Doubtful	NR	NR	NR	NR	NR	NR
Galiana et al. (PSC) [30]	Very good	Very good	NR	NR	NR	Adequate	NR	NR
Kohli et al. (SCA) [31]	NR	Doubtful	NR	NR	NR	NR	NR	NR
Zeb et al. (MSC) [32]	NR	Very good	NR	NR	NR	NR	NR	NR
Yang et al. (MSC) [33]	Very good	Very good	Inadequate	Inadequate	NR	NR	NR	NR
Ahmadi et al. (SAQ) [14]	NR	Doubtful	Inadequate	NR	NR	NR	NR	NR

Scores for methodological quality using COSMIN Risk of Bias Checklist: very good, adequate, doubtful, inadequate; NR: Not reported

### Quality of measurement properties of scales

The measurement properties of the scales in each study were rated according to updated criteria for good measurement properties. All the scales were reported in one study except for MSC, reported in two studies [32, 33]. The results of these two studies are qualitatively summarized. In Zeb et al.‘s [32] study, only the internal consistency of the MSC was calculated in 50 nurses, which obtained a very good score from a methodological quality. But Yang et al. [33], translate the MSC into Chinese and validate its reliability and validity among 510 hospice nurses. In terms of internal consistency, Yang’s study was consistent with Zeb et al.‘s. In terms of other measurement properties, considering that the quality of Yang’s study was higher than that of Zeb et al.‘s study, the overall rating of the measurement properties of the MSC scale was again given based on Yang’s study. The overall rating of the quality of the measurement properties is in Table 5.

**Table 5:** Summary of findings (Quality of measurement properties results)

Scales	Structural validity		Internal consistency		Cross-cultural validity (Measurement invariant)			Reliability (Test-retest)		Construct validity (Hypotheses testing)	
	Overall rating ^1^	QoE ^2^	Overall rating^1^	QoE ^2^	Overall rating^1^	QoE^2^	Overall rating^1^		QoE^2^	Overall rating^1^	QoE^2^
SCBN	?	NA	?	Low	?	NA	?		NA	?	NA
PSC	-	Moderate	-	Moderate	?	NA	?		NA	Results in line with 5 hypo’s (5+)	Low
SCA	?	NA	?	Very low	?	NA	?		NA	?	NA
MSC	+	High	+	High	?	Very low	?		Very low	?	NA
SAQ	?	NA	?	Very low	?	Very low	?		NA	?	NA

^1^Overall rating of quality of measurement properties (Rating: sufficient (+), insufficient (–), inconsistent (±), or indeterminate (?)); ^2^Quality of the evidence, using a modified GRADE approach (Rating: high, moderate, low, very low evidence); NA: Not assessed

#### Structural validity

In the PSC, it is insufficient (CFI = 0.91, RMSEA = 0.1 SRMR = 0.09), and in MSC (RMSEA = 0.044 0.1, CFI = 0.96) is sufficient. In other scales were not reported.

#### Internal consistency

In the MSC, it is sufficient, because, there is high evidence for sufficient construct validity and Cronbach’s alpha is above 0.7. In the PSC, it is insufficient. Cronbach’s alpha in dimensions of PSC is 0.62 and 0.57. In the rest of the scales, an undetermined score was given because the construct validity has not been determined.

#### Cross-cultural validity

The score of scales of MSC and Self-Assessment Questionnaire (SAQ) is indeterminate because, in these scales, translation and re-translation (backward) process for cross‐cultural validity has been carried out, but no multiple group factor analysis or DIF analysis was performed. In other scales, cross‐cultural validity was not reported.

#### Reliability

Because Intra-Class Correlation (ICC) or weighted Kappa was not checked in any of the scales, an indeterminate score was considered for all scales.

#### Construct validity

This property in PSC is sufficient, and all five determined hypotheses were confirmed.

### Selecting the Scale

None of the scales were categorized as A. Based on the COSMIN methodology, for the recommendation of a scale, it should have a minimum of low-level evidence for internal consistency and any level of content validity [22]. So, considering that the content validity of Self-Care Behaviors of Nurses Scale (SCBN), Self-Care Assessment Worksheet (SCA), and SAQ was not assessed, it is impossible to decide in which category of recommendation they fall.

The scales of MSC and PSC were classified in the category of B and can be temporarily used, but they require further research to assess. Based on COSMIN methodology, when scales are categorized as ‘B’, the one with the best evidence for content validity could be the one to be provisionally recommended for use until further evidence is provided. However, the overall rating of the content validity scales of MSC and PSC is similar. Of course, considering that MSC is designed for people over 18 years old, it is suggested that PSC made for professionals, including nurses and nursing students, should be used temporarily.

## Discussion

This systematic review evaluated the measurement properties of self-care scales for nurses to identify the best available scales. Based on COSMIN methodology, the results showed that none of the scales had methodological quality with a rating of very good and sufficient high-quality evidence for all measurement properties.

All the scales examined in this study were based on the CTT theory, and none used the Item Response Theory (IRT). Although it is more difficult to use IRT to examine the psychometric properties of scales than CTT, IRT is a superior method for providing a complete psychometric evaluation of a scale intended for intervention studies and clinical trials [34] and is more sensitive to cross-sectional changes in health over time [35].

Two studies that reported cross-cultural validity had low methodological quality for this property with insufficient or indeterminate results. Cross-cultural validity shows whether items of a translated or culturally adapted scale properly reveal the originally developed scale [22]. Therefore, the results of the studies that used these translated scales are not reliable, and it is necessary to evaluate this property in future studies.

In this study, content validity was examined in only two scales, which had inconsistent ratings with low-quality evidence. At the same time, content validity is the most important measurement property. In the COSMIN guideline, special attention has been paid to it and showed the degree to which the content of a scale is an adequate reflection of the construct [22]. Lack of content validity can affect all other measurement properties. For example, it may decrease internal consistency, structural validity, and interpretability [26]. In addition, this property is an important condition for providing evidence-based recommendations for selecting scales in systematic reviews. As regards, this property is often not explained in detail in studies or is not done in principle; in other systematic studies on the measurement properties of scales, there were many challenges in examining this property [36–38].

Among the six scales examined, only PSC was specifically designed to examine self-care in nurses, and the rest were general self-care scales. It should be noted that general and non-specific scales have low sensitivity for measuring specific cases and cannot show changes in a specific population because the characteristics and subcultures of the reference population are not included in determining their items [39]. Of course, the dimensions and items of PSC are also completely general. As mentioned in the results section, item generation has not been done using the target population and during a proper qualitative study in these scales. Indeed, nurses have self-care needs that are appropriate to their professional activity. It is expected that the special self-care scale for nurses can evaluate their self-care behaviors against occupational hazards. In addition, in Mills’s definition, self-care is divided into two general dimensions (personal and professional) [40].

Personal dimensions are completely general and include physical, mental, spiritual, social, and recreational. But regarding professional self-care, depending on the work conditions, the definitions, dimensions, and performance strategies recommended for implementing self-care are different [41]. Professional self-care involves engaging in practices that ensure balance and effectiveness in the professional role [40]. For example, the psychological dimension (resilience strategies against work stress) [41], the social dimension (strategies to strengthen interpersonal support in the workplace) [42], the balance dimension (managing work and time pressures and maintaining boundaries between work and family life) [43], growth dimension (strategies to advance professional life, skills, and professional knowledge) [41] and energy generation dimension (activities to preserve energy, hope is in the work environment) [40]. In this study, only SCA had a professional self-care dimension. Of course, this scale was not recommended due to the lack of content validity report and incomplete measurement properties [31].

In examining the psychometric properties of the investigated scales, characteristics of criterion validity, measurement error, responsiveness, feasibility, and interpretability were not measured and reported in the scales. These are important properties and should not be overlooked. In line with this finding, in the review and qualitative appraisal of self-care scales in healthy adults with COSMIN guidelines, measurement error and responsiveness were not reported in the scales [17]. Also, in the results of other systematic review studies on measurement properties scales with COSMIN guidelines, these characteristics were also not measured [24, 44].

One component of the validity domain is criterion validity, which is the extent to which the scores on a scale adequately reflect (or predictor of) a criterion or “gold standard. Sometimes gold standards are expensive, invasive, and have limited access. In this case, a high correlation scale with this standard can be a good alternative [45]. Although criterion validity is considered a strength of a scale, it was not calculated in the scales examined in this study. In fact, not all scales can be validated using a criterion approach because there is not always a valid and reliable gold standard to use as the criterion [25]. Self-care in nurses is a complex and multifaceted concept (with dimensions of physical, psychological, spiritual, social, professional, etc.). It isn’t easy to choose a gold standard for this concept. In this regard, Matarez, who examined self-care scales in healthy people with the COSMIN guideline, also had the same opinion and did not examine this criterion [17]. In these cases, it is suggested that researchers rely on hypothesis-testing construct validity instead of criterion validity [45].

Measurement error was another important property that was not reported in any of the investigated scales. Based on CTT theory, measurement error, to some extent, is introduced into all measurement scales that randomly or systematically limit the degree of precision in estimating the actual scores from observed scores. Measurement error is the main threat to the reliability of the scale. Since reliability is a necessary prerequisite for validity, measurement error also affects validity [46]. Considering that the goal of all scales is to achieve correct values, therefore, the measurement error of the scales should be determined by calculating minimal important change (MIC) or smallest detectable change (SDC) [25]. Therefore, it is necessary to investigate and report the measurement error of these scales in future studies so that these scales can be used more reliably.

Responsiveness was also a very important and neglected criterion in the examined scales. This property is the ability of a scale to detect change over time in a construct and shows whether a change score truly captures a change in the construct. There are similarities and overlaps between responsiveness and validity (construct and criterion). For this reason, some scale developers do not support using the term responsiveness as a separate measurement property [45]. But in COSMIN’s guideline, to pay more attention to this property, it has been reported independently of validity [22]. This property is sensitive to treatment and is beneficial for healthcare professionals profoundly concerned with measuring change. However, some scale developers don’t examine this property because it’s time-consuming [45]. Considering that self-care is amenable to change in a person. Therefore, the measurement scale of this construct should have the property of responsiveness. Responsiveness relies on ongoing evidence building [45]. Therefore, researchers can check the responsiveness property of the scales examined in this study in the future.

Although feasibility and interpretability are not measurement properties, they are two important characteristics that show the usefulness of any scale [45]. These two characteristics were not reported in any of the investigated scales. Indeed, by using interpretability, we can give qualitative meaning (that is, clinical or commonly understood connotations) to quantitative scores or changes in scores on a scale [25]. Also, due to time and cost limitations in research, information on the feasibility or ease of application of the scale, such as cost and length of the scale, completion time, etc. will help researchers to choose the right scale for their situation [25]. Therefore, the designers of these scales must provide information related to feasibility and interpretability in a way.

### Strengths and limitations

To our knowledge, no systematic review has been conducted that has examined the measurement properties of self-care scales in nurses in a detailed and comprehensive manner. Considering that the criticism of the scales using the COSMIN guideline is a very specialized, time-consuming, and difficult, the data analysis was done as a teamwork. One of the strengths of this study was the presence of a highly experienced scale design specialist in the research team who oversaw the data analysis (AE).

This study was associated with limitations including: in this study, only articles in Farsi and English were examined due to the lack of proficiency of the authors in other languages, the search for articles was done from four main and reliable electronic databases, and access to other databases was limited for the researcher, so the Google Scholar search engine was used to complete the search and access more articles, and the list references of the included articles were also checked. Some included studies had reported incomplete data in the methodology or results related to measurement properties, which caused either those properties not to be examined or to receive a low score. Despite the researcher’s numerous follow-ups, the original text of some of the investigated scales could not be accessed, and this limitation caused the content validity of these instruments not to be checked. Because according to the COSMIN methodology, the items on the scale should have been studied by researchers and scored. Although the scales’ measurement properties were analyzed carefully using the COSMIN guideline, this process is partly subjective, especially for the content validity, which the researcher must also rate. Therefore, data analysis was done separately by two authors, and in case of ambiguity, the third author was consulted.

## Conclusion and implications

In this systematic review study, none of the scales were placed in category A, and using them is strongly not recommended. Only PSC was placed in category B and is temporarily recommended until its quality is assessed in future studies. This study identified the gaps and defects of the examined scales. It is recommended that more robust studies be conducted to investigate the measurement properties of these scales, especially in the field of properties that have not been assessed in any of them (such as measurement error). Finally, according to the research team’s opinion that the content of any of these scales does not meet all the self-care needs of nurses, it is necessary to design a specialized scale for nurses. A scale that uses a strong qualitative study with the participation of nurses from different departments of the hospital to items generation. This study has evaluated the scales step by step based on COSMIN guidelines, so it can be a training guide for those who intend to design or critique scales.

It is necessary to discover the weakness of self-care in nurses with a specific, valid, and reliable scale because the lack of self-care leads to decreased quality of care and patient safety. Data from this specific scale can be provided to health policy-makers to help improve nurses’ self-care ability by designing comprehensive, practical, and cost-effective programs and creating a suitable and facilitating platform in clinical environments.

### Electronic supplementary material

Below is the link to the electronic supplementary material.

Additional file 1: The search syntax of each database

## Data Availability

Datasets are available through the corresponding author upon reasonable request.

## Abbreviations

COSMIN: The Consensus-based Standards for the Selection of Health Measurement Instruments
PRISMA: Preferred Reporting Items for Systematic Reviews and Meta-Analyses; Patient Reported Outcome Measures (PROMs)
GRADE: Grading of Recommendations Assessment, Development, and Evaluation
PSC: Professional Self-Care Scale
SCBN: Self-care Behaviors of Nurses Scale
SCA: Self-care Assessment Worksheet
MSC: Mindful Self-care Scale
SAQ: Self-Assessment Questionnaire
QoE: Quality of the Evidence
CTT: Classical Test Theory
NR: Not Reported
MIC: Minimal Important Change
SDC: Smallest Detectable Change
LOA: Limits of Agreement
ICC: Intra-Class Correlation
SD: Standard Deviation
CFA: Confirmatory Factor Analysis
EFA: Exploratory Factor Analysis
KMO: Kaiser-Meyer-Oklin
RMSEA: Root Mean Square Error of Approximation
CFI: Confirmatory Fit Index
TLI: Tucker-Lewis Index
NFI: Normed Fit Index
PNFI: Parsimonious Normed-of-Fit Index
CRIs: Composite Reliability Indexes
SOC: Sense of Coherence Questionnaire
CR: Critical Ratio
GFI: Goodness-of Fit Index
AGFI: Adjusted Goodness-of-Fit Index
PGFI: Parsimonious Goodness-of-Fit Index
IFI: Incremental Fit Index
CVR: Content Validity Ratio
CVI: Content Validity Index
