# Supplementary material for: A systematic review of the measurement properties of self-care scales in nurses
Source: BMC Nurs. 2023 Aug 28;22:288. doi: 10.1186/s12912-023-01450-2 (PMC10463637; doi:10.1186/s12912-023-01450-2)
Supplement: Supplementary file 1 — Additional file 1: The search syntax of each database [file 12912_2023_1450_MOESM1_ESM.docx]

Additional table 1. The search syntax of each database

| Database | Search terms | Number of articles |
| --- | --- | --- |
| PubMed | (“Self care”[tiab] OR “Self-care”[tiab] OR “self care deficit”[tiab] OR “self care agency”[tiab] OR “self care requisite*”[tiab] OR “Personal self-care”[tiab] OR “Organizational self-care”[tiab] OR “Care of self”[tiab] OR “Care of the self”[tiab] OR “Care for themselves”[tiab] OR “Mindful self-care”[tiab] OR “themselves-care”[tiab] OR “Professional self-care”[tiab]) AND (Nurs*[tiab] OR “Nursing staff”[tiab] OR “Nursing personnel*”[tiab] OR “Nursing Assistant*”[tiab]) AND (Scale*[tiab] OR Instrument*[tiab] OR Tool*[tiab] OR Validation*[tiab] OR Psychometric[tiab] OR inventory[tiab] OR Checklist*[tiab] OR Questionnaire*[tiab] OR Assessment[tiab] OR Measure*[tiab] OR Evaluation[tiab]) | 2493 |
| Scopus | TITLE-ABS(“Self care” OR “Self-care” OR “self care deficit” OR “self care agency” OR “self care requisite*” OR “Personal self-care” OR “Organizational self-care” OR “Care of self” OR “Care of the self” OR “Care for themselves” OR “Mindful self-care” OR “themselves-care” OR “Professional self-care”) AND TITLE-ABS(Nurs* OR “Nursing staff” OR “Nursing personnel*” OR “Nursing Assistant*”) AND TITLE-ABS(Scale* OR Instrument* OR Tool* OR Validation* OR Psychometric OR inventory OR Checklist* OR Questionnaire* OR Assessment OR Measure* OR Evaluation) | 3075 |
| Web of science | TS=(“Self care” OR “Self-care” OR “self care deficit” OR “self care agency” OR “self care requisite*” OR “Personal self-care” OR “Organizational self-care” OR “Care of self” OR “Care of the self” OR “Care for themselves” OR “Mindful self-care” OR “themselves-care” OR “Professional self-care”) AND TS=(Nurs* OR “Nursing staff” OR “Nursing personnel*” OR “Nursing Assistant*”) AND TS=(Scale* OR Instrument* OR Tool* OR Validation* OR Psychometric OR inventory OR Checklist* OR Questionnaire* OR Assessment OR Measure* OR Evaluation) | 2312 |
| ProQuest | TI,AB,SU(“Self care” OR “Self-care” OR “self care deficit” OR “self care agency” OR “self care requisite*” OR “Personal self-care” OR “Organizational self-care” OR “Care of self” OR “Care of the self” OR “Care for themselves” OR “Mindful self-care” OR “themselves-care” OR “Professional self-care”) AND TI,AB,SU(Nurs* OR “Nursing staff” OR “Nursing personnel*” OR “Nursing Assistant*”) AND TI,AB,SU(Scale* OR Instrument* OR Tool* OR Validation* OR Psychometric OR inventory OR Checklist* OR Questionnaire* OR Assessment OR Measure* OR Evaluation) | 647 |
| Google Scholar | All in title: self care, nurse, nursing, Scale, Instrument, Tool, Validation, Psychometric, inventory, Checklist, Questionnaire, Assessment, Measure, Evaluation | 67 |
